# Awareness and attitudes of pre-exposure prophylaxis for HIV prevention among physicians in Guatemala: Implications for country-wide implementation

**DOI:** 10.1371/journal.pone.0173057

**Published:** 2017-03-03

**Authors:** Ian Ross, Carlos Mejia, Johanna Melendez, Philip A. Chan, Amy C. Nunn, William Powderly, Katherine Goodenberger, Jingxia Liu, Kenneth H. Mayer, Rupa R. Patel

**Affiliations:** 1 Division of Infectious Diseases, Washington University School of Medicine, St. Louis, Missouri, United States of America; 2 Division of Infectious Diseases, Roosevelt Hospital, Guatemala City, Guatemala; 3 Division of Infectious Diseases, Brown University, Providence, Rhode Island, United States of America; 4 Department of Behavioral and Social Sciences, Brown University School of Public Health, Providence, Rhode Island, United States of America; 5 Division of Public Health Sciences, Washington University School of Medicine, St. Louis, Missouri, United States of America; 6 Division of Infectious Diseases, Beth Israel Deaconess Medical Center, Boston, Massachusetts, United States of America; 7 Division of Infectious Diseases, Harvard Medical School, Boston, Massachusetts, United States of America; 8 The Fenway Institute, Fenway Health, Boston, Massachusetts, United States of America; University of Toronto, CANADA

## Abstract

**Introduction:**

HIV continues to be a major health concern with approximately 2.1 million new infections occurring worldwide in 2015. In Central America, Guatemala had the highest incident number of HIV infections (3,700) in 2015. Antiretroviral pre-exposure prophylaxis (PrEP) was recently recommended by the World Health Organization (WHO) as an efficacious intervention to prevent HIV transmission. PrEP implementation efforts are underway in Guatemala and success will require providers that are knowledgeable and willing to prescribe PrEP. We sought to explore current PrEP awareness and prescribing attitudes among Guatemalan physicians in order to inform future PrEP implementation efforts.

**Methods:**

We conducted a cross-sectional survey of adult internal medicine physicians at the main teaching hospital in Guatemala City in March 2015. The survey included demographics, medical specialty, years of HIV patient care, PrEP awareness, willingness to prescribe PrEP, previous experience with post-exposure prophylaxis, and concerns about PrEP. The primary outcome was willingness to prescribe PrEP, which was assessed using a 5-point Likert scale for different at-risk population scenarios. Univariate and multivariate logistic regression was performed to identify predictors for willingness to prescribe PrEP.

**Results:**

Eighty-seven physicians completed the survey; 66% were male, 64% were internal medicine residency trainees, and 10% were infectious disease (ID) specialists. Sixty-nine percent of physicians were PrEP aware, of which 9% had previously prescribed PrEP. Most (87%) of respondents were willing to prescribe PrEP to men who have sex with men (MSM), sex workers, injection drug users, or HIV-uninfected persons having known HIV-positive sexual partners. Concerns regarding PrEP included development of resistance (92%), risk compensation (90%), and cost (64%). Univariate logistic regression showed that younger age, being a resident trainee, and being a non-ID specialist were significant predictors for willingness to prescribe PrEP. In multivariate logistic regression, being a non-ID specialist was a significant predictor.

**Conclusions:**

Guatemalan physicians at an urban public hospital were PrEP aware and willing to prescribe, but few have actually done so yet. Future education programs should address the concerns identified, including the low potential for the development of antiretroviral resistance. These findings can aid PrEP implementation efforts in Guatemala.

## Introduction

Human immunodeficiency virus (HIV) continues to be a major cause of morbidity and mortality worldwide. Globally, approximately 2.1 million people were newly infected with HIV in 2015 [1]. Given the large number of new HIV infections each year, preventing transmission remains a major goal for clinical care and research. Sexual risk reduction programs [2], condom use [3], and routine screening for and treatment of sexually transmitted infections (STIs) [4,5] serve as HIV prevention strategies that have impacted global transmission. However, these interventions have not been able to end the epidemic. Clinical trials have demonstrated efficacy of antiretroviral pre-exposure prophylaxis (PrEP) in preventing HIV acquisition in medication-adherent, at-risk populations [6–9]. Currently available PrEP is a once daily oral medication that is tenofovir disoproxil fumarate (TDF) and emtricitabine (FTC) or TDF alone. The World Health Organization (WHO) recently expanded its guidelines for use of PrEP for HIV prevention in resource-limited settings and among at-risk populations [10]. In order to implement these guidelines and effectively deliver PrEP to at-risk populations, health care providers must be both knowledgeable about and willing to prescribe PrEP. However, provider acceptability studies have mostly been conducted at PrEP research study sites or within North America [11–17]. Understanding provider concerns and barriers to PrEP prescribing in specific contexts (e.g. different geographical and cultural settings) has the potential to expand PrEP coverage among key populations to accomplish the overall goal of HIV incidence reduction regionally and worldwide.

Central America accounted for approximately 8,700 new HIV infections worldwide in 2015 [1]. Guatemala had the highest incidence within the region with 3,700 infections per year. This figure is more than three times the number of new infections in any other Central American country [1]. Estimated national HIV seroprevalence among adults aged 15 to 49 years was 0.6% in 2015. However, prevalence was higher in key populations including men who have sex with men (MSM) (12%) and sex workers in urban areas (4–15%) [18]. A large portion of HIV infections occurred in Metropolitan Region, one of eight municipal country divisions and where Guatemala City is located [19]. PrEP holds promise in curbing HIV incidence among key populations in urban Guatemala. Therefore, determining PrEP awareness, acceptability, and concerns among providers in this region can guide PrEP education and implementation programs.

Currently, three Joint United Nations Programme on HIV and AIDS discussions have been held in Guatemala surrounding PrEP implementation since November 2015 [C.R. Mejia, personal communication, January 14, 2016]. There has been no government-issued policy or guidelines released for PrEP prescribing nor a medication regulatory application filed to the local Ministry of Health for the use of TDF or TDF/FTC for PrEP. TDF/FTC has been approved for HIV treatment in Guatemala and has been used for first-line treatment since 2008. TDF/FTC is currently obtained from Indian manufacturers. PrEP is currently being prescribed in a limited fashion through the unregulated private sector and mainly to serodiscordant couples [C.R. Mejia, personal communication, January 14, 2016].

Previously reported challenges in PrEP implementation in other countries include lack of access to healthcare [20], access to antiretrovirals [21,22], medication regulatory approval [23], PrEP awareness among key populations [21], providers’ barriers to prescribing (including concerns about drug resistant viral strains, riskier sexual behavior, and diversion of limited financial resources) [24], and plans to cover medication costs for patients who cannot afford them [22].

In light of the WHO guidelines and ongoing discussions surrounding PrEP implementation in Guatemala, we conducted a study to assess PrEP awareness, willingness to prescribe, and concerns among physicians at a major public hospital in Guatemala City.

## Methods

### Sample population

We conducted a cross-sectional quantitative survey among adult internal medicine residency trainees, infectious diseases (ID) fellowship trainees, and faculty (both specialists and non-specialists) at Roosevelt Hospital in Guatemala City, Guatemala. Roosevelt Hospital is a public hospital and is affiliated with an internal medicine residency academic program. The 3-year residency program has 70 residents per year. The hospital system also has one of the largest outpatient HIV clinics in Guatemala, which provides care to over 4,100 rural and urban patients from all over the country.

### Data collection

The study was conducted over four weeks from March 7, 2015 to April 7, 2015. The self-administered questionnaire included age, gender, residency trainee status, and specialty practiced among faculty (e.g. ID, cardiology). ID fellows were categorized as ID specialists in this study. Non-ID specialties included cardiology, gastroenterology, and critical care. The questionnaire also asked about PrEP awareness and willingness to prescribe, previous use of post-exposure prophylaxis, concerns about PrEP, knowledge of HIV transmission (e.g. anal sex), HIV prevention methods (e.g. condom use) discussed for the care of current at-risk patients, and preferences for future PrEP education. At the time of this study, there were no formalized Ministry of Health guidelines for PrEP use in Guatemala. There was a National Guideline for Antiretroviral Treatment 2013 and a local Roosevelt Hospital protocol for prescribing occupational post-exposure prophylaxis for healthcare workers and for victims of sexual assault [C.R. Mejia, personal communication, May 8, 2016]. Study eligibility included being 18 years or older and staff at Roosevelt Hospital. No compensation was provided for survey completion. Providers were recruited at lectures and other meetings held at the hospital. The surveys were translated into Spanish. Consent was obtained orally and was not recorded. The research presented no more than minimal risk and obtained minimal demographic information. Taking the survey was voluntary and procedures were explained to participants beforehand. To ensure confidentiality, we did not obtain written consent and did not document participant names. This consent procedure and study were approved by the Washington University in St. Louis Institutional Review Board (IRB) and the Roosevelt Hospital IRB.

### Outcomes

The primary outcome was willingness to prescribe PrEP for at least one at-risk population. This outcome was assessed using a Likert scale in 5 different at-risk population scenarios: MSM who report regular condom use with anal sex, MSM who report condomless anal sex, persons who inject drugs (PWID), MSM or heterosexual person in a serodiscordant relationship, and sex workers (male or female). The Likert scale used was: 1) would not prescribe, 2) probably would not prescribe, 3) neutral/undecided, 4) probably would prescribe, and 5) would prescribe PrEP. A binary measure of “willing” (answers 4 or 5) versus “unwilling” (answers 1–3) was created. Willingness to prescribe was analyzed for each at-risk population scenario and then combined for the primary outcome of at least one at-risk group.

The secondary outcome was PrEP awareness and was assessed by reporting: 1) have never heard of PrEP, 2) know a little, 3) have read the studies, or 4) have previously prescribed PrEP. PrEP awareness was defined as reporting 2–4.

Concerns with PrEP prescribing were determined by using a Likert scale: 1) not at all concerned, 2) somewhat concerned, 3) concerned, 4) very concerned, and 5) it would prevent me from prescribing PrEP. A binary measure of “concerned” was created for answer choices 3–5. Topics assessed were medication toxicity, increase in high-risk sexual behavior or risk compensation, development of antiviral resistance, potential loss of government funding for other HIV prevention methods, medication costs, and the need for regular follow up visits.

Providers were also asked their willingness to prescribe PrEP in the following situations: 1) efficacy rates were greater than 90% for all at-risk groups, 2) government-endorsed guidelines were available, 3) medication costs were lower, and 4) educational workshops were provided. At the time of this study, oral TDF/FTC was 92% effective for MSM and transgender females when there were detectable blood tenofovir levels [6], but efficacy at 90% or above was not found for PWID [9] or heterosexual females [8,25].

Lastly, physicians were asked (“yes” or “no”) if they desired additional data to inform PrEP prescribing. Data requested included: 1) evidence of efficacy, 2) patient acceptability, 3) other countries’ use of PrEP, 4) medication costs, and 5) potential healthcare cost savings with PrEP.

### Statistical analysis

Descriptive statistics were used to summarize the provider demographics and Likert scale categorization.

For the outcomes of PrEP awareness and willingness to prescribe, univariate and multivariate logistic regression analysis was performed. The independent variables included age, gender, resident trainee, ID specialist, and prior post-exposure prophylaxis prescribing. All the tests were two-sided and the significance level was set at 0.05. The statistical package SAS 9.4 was used (SAS Institute Inc., Cary, NC).

## Results

Eighty-seven providers participated in the survey. Participation was 100%. Respondent demographics are displayed in Table 1.

**Table 1:** Physician demographics (N = 87).

Variable	N (%)
Median Age (Interquartile Range) (Years)	28 (26–38)
Gender	
Male	56 (66)
Female	29 (34)
Resident trainee	
No	30 (36)
Yes	53 (64)
ID specialist	
No	75 (90)
Yes	8 (10)
Prior post-exposure prophylaxis prescribing	
No	59 (68)
Yes	28 (32)

The denominator for the percentages is the sum of participants across all categories excluding missing values.

Of the participants: median age was 28 years, age range was 24–72 years, 66% were male, 64% were residents, and 10% were ID specialists. When assessing HIV transmission knowledge, 83% of respondents correctly identified anal sex as the highest risk for HIV transmission compared to penile-vaginal and oral intercourse. Current HIV prevention practices recommended to patients included discussing consistent condom use (85%), screening for at-risk sexual behaviors (84%), screening for PWID (84%), providing behavioral counseling (72%), screening for STIs (64%), regular HIV testing (32%), and promoting abstinence (33%). Thirty-two percent of respondents had previously prescribed occupational or non-occupational post-exposure prophylaxis, of which 18 physicians reported prescribing to a sexual assault victim.

**Fig 1:**
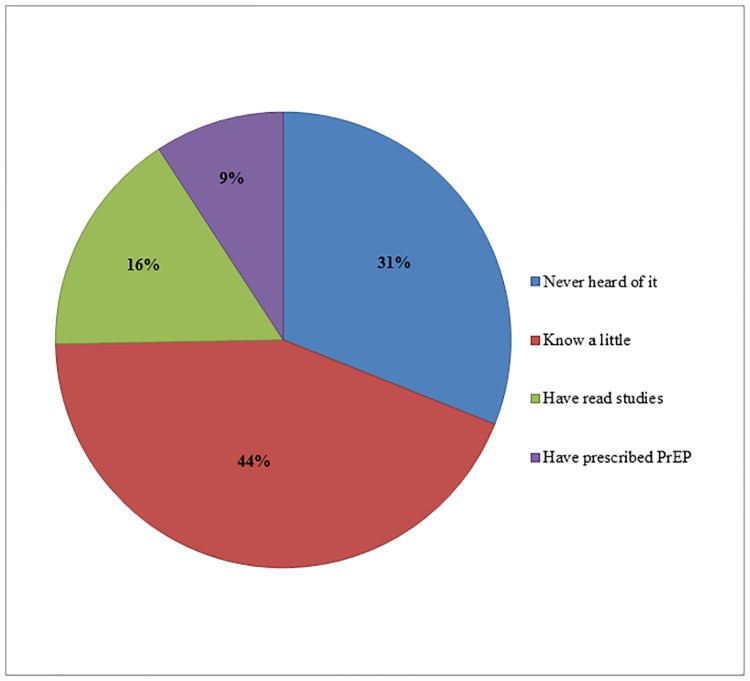
PrEP awareness among internal medicine physicians in Guatemala City (N = 87). PrEP awareness was defined as either knowing a little, having read studies, or having prescribed PrEP.

PrEP awareness among providers was 69% (Fig 1). Of those aware, 9% had previously prescribed PrEP.

**Fig 2:**
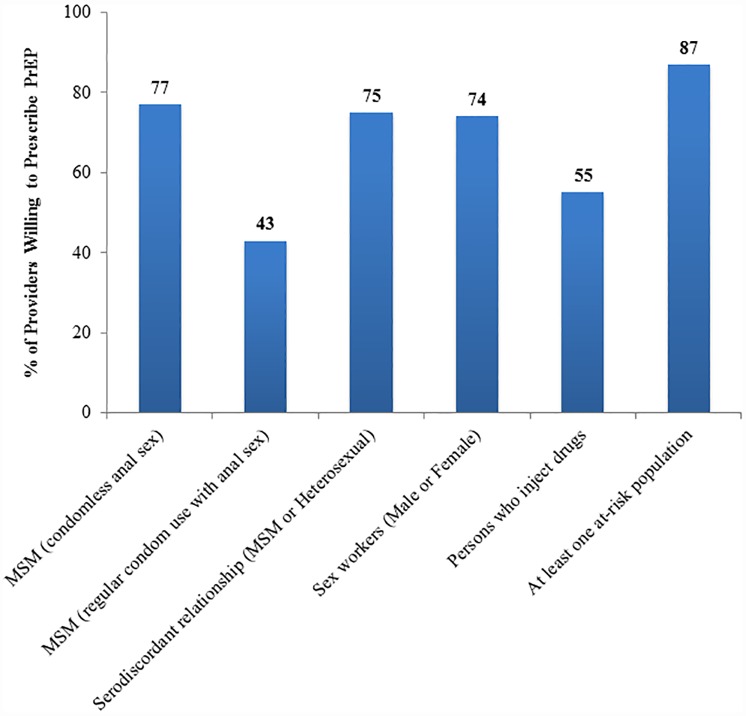
Physician willingness to prescribe PrEP for at-risk population scenarios. Physician willingness to prescribe was defined as somewhat likely, likely or very likely to prescribe PrEP based on a Likert scale response. Abbreviations: men who have sex with men (MSM).

Most (87%) indicated that they were willing to prescribe PrEP to at least one of the five at-risk populations (Fig 2). Providers were most willing to prescribe PrEP to MSM who report condomless anal sex (77%).

Correlates of willingness to prescribe PrEP for at least one at-risk population and PrEP awareness were explored. Univariate logistic regression revealed age, resident training status, and being an ID specialist as significant predictors of willingness to prescribe PrEP (Table 2). Older providers (OR = 0.93, 95% CI: 0.86–0.98; p = 0.01) and non-ID specialists (OR = 0.09, 95% CI: 0.02–0.47; p = 0.004) were less willing to prescribe PrEP. Resident trainees were more likely to prescribe PrEP (OR = 5.22, 95% CI: 1.23–22.20; p = 0.03). However, PrEP awareness was not a significant predictor of willingness to prescribe (OR = 0.44, 95% CI: 0.09–2.18; p = 0.31). Multivariate regression analysis revealed being a non-ID specialist was a significant predictor of willingness to prescribe PrEP when adjusting for age, gender, resident trainee status, prior post-exposure prophylaxis prescribing, and PrEP awareness (OR = 0.07, 95% CI: 0.005–0.935; p = 0.04) (Table 2).

**Table 2:** Univariate logistic regression analysis for willingness to wrescribe PrEP among urban Guatemalan physicians (N = 82).

Characteristic	Crude OR (95% CI)	P value	Adjusted OR (95% CI)	P value
Age	0.93 (0.86, 0.98)	**0.01**	0.95 (0.86, 1.05)	0.30
Gender		0.44		0.12
Male	1.0		1.0	
Female	0.60 (0.17, 2.18)		0.23 (0.04, 1.46)	
Resident trainee		**0.03**		0.89
No	1.0		1.0	
Yes	5.22 (1.23, 22.20)		0.83 (0.06, 11.78)	
ID specialist		**0.004**		**0.04**
No	1.0		1.0	
Yes	0.09 (0.02, 0.47)		0.07 (0.005, 0.935)	
Prior post-exposure prophylaxis prescribing		0.67		0.12
No	1.0		1.0	
Yes	1.36 (0.33, 5.60)		6.66 (0.62, 71.40)	
PrEP awareness		0.31		0.25
No	1.0		1.0	
Yes	0.44 (0.09, 2.18)		0.24 (0.02, 2.77)	

There were no independent predictors for PrEP awareness in univariate or multivariate analysis (Table 3). All 25 persons who were PrEP unaware were non-ID specialists.

**Table 3:** Univariate logistic regression analysis for PrEP awareness among urban Guatemalan physicians (N = 87).

Characteristic	Crude OR (95% CI)	P value	Adjusted OR (95% CI)	P value
Age	1.01 (0.97, 1.06)	0.61	0.97 (0.87, 1.06)	0.52
Gender		0.38		0.25
Male	1.0		1.0	
Female	0.65 (0.25, 1.69)		0.53 (0.18, 1.56)	
Resident trainee		0.05		0.10
No	1.0		1.0	
Yes	0.33 (0.11, 1.00)		0.19 (0.03, 1.38)	
Prior post-exposure prophylaxis prescribing		0.07		0.08
No	1.0		1.0	
Yes	2.74 (0.91, 8.23)		2.81 (0.87, 9.02)	

**Fig 3:**
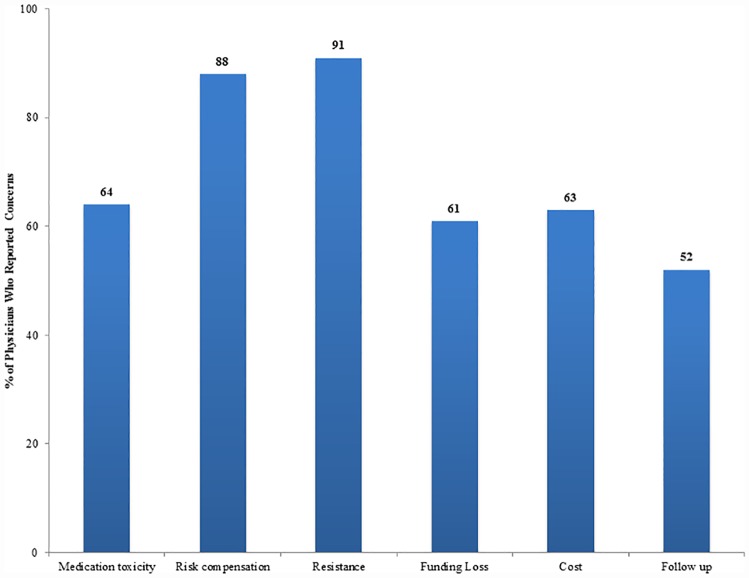
Physician PrEP prescribing concerns in Guatemala City. Physician concern was defined as concerned, very concerned, or it would prevent me from prescribing PrEP based on a Likert scale response.

Physician PrEP prescribing concerns included the development of antiretroviral drug resistance (91%), risk compensation (88%), medication toxicity (64%), medication costs (63%), potential loss of government funding for other HIV prevention measures (61%), and the need for regular follow-up visits (52%) (Fig 3).

Of the hypothetical implementation-related situations in which willingness to prescribe PrEP would increase, the following was reported: efficacy above 90% for all at-risk populations (75%), government-endorsed guidelines (57%), lower medication costs (60%), and available education workshops (64%).

Lastly, additional data that providers desired in order to increase PrEP utilization were: PrEP efficacy (85%), patient acceptability (56%), other countries’ use of PrEP (64%), medication costs (67%), and potential healthcare cost savings (53%).

## Discussion

This study among urban-based Guatemalan physicians revealed high PrEP awareness (69%) and willingness to prescribe (87%). This is the first study conducted in Guatemala regarding PrEP provider awareness and acceptability.

The study was performed prior to the WHO guidelines for PrEP 2015 [10] and can provide timely insight on national PrEP implementation programs for key populations. Specifically, our results can inform local dissemination efforts among physicians and can provide insight on the real-world barriers for PrEP prescribing in Guatemala. Early adopters of PrEP prescribing may include ID specialists, prior post-exposure prophylaxis prescribers, and those about to complete residency. Furthermore, 64% were willing to prescribe if they had access to provider workshops, which supports the need to move towards a national provider training program as the first steps of country-wide PrEP implementation.

Our study population’s level of PrEP awareness was higher than that reported in Lima, Peru (57.5%) [13], but lower than that conducted among North American HIV providers (90%) [15]. The time period and PrEP awareness definitions differ among prior studies and may account for differences in the prevalence of PrEP awareness found. For example, Tang et al. reported Peruvian provider attitudes surrounding PrEP in 2012, two years after the Pre-exposure Prophylaxis Initiative (iPrEx) results were published [6], where participants were asked whether they had heard of PrEP as the use of oral antiretrovirals prior to exposure [13]. Healthcare workers, 40% of whom were physicians, were more aware of PrEP if their role was to prescribe antiretrovirals, had cared for at least 50 MSM or at least 50 HIV-infected patients, or were ID specialists. Provider prescribing concerns were the lack of government-issued PrEP guidelines, risk compensation, and the development of resistance, which were also found in our study [13]. In contrast, only 10% of our sample was ID physicians, who may have had more exposure to at-risk populations and HIV care and thus possibly more PrEP awareness. This sample represents physicians from all internal medicine backgrounds and may reflect PrEP awareness generalizable to a physician population in an urban Guatemalan area. The Peruvian study by Tang et al. was conducted in the context of iPrEX at two Peruvian sites, Lima and Iquitos. Having a clinical trial in the local area may have contributed to such high levels of PrEP awareness despite not having any formal guidelines in place at that time. We postulate that the presence of the July 2014 WHO guidelines [26] recommending PrEP for MSM and the presence of iPrEX in Latin America may have contributed to our sample’s high PrEP awareness, based on our study definition. Moreover, Roosevelt Hospital physicians may have had more exposure to HIV care, regardless of specialty, because it is affiliated with one of the largest outpatient HIV clinics in Guatemala. The department also held HIV education programs, which included one academic journal club on PrEP.

Tellalian et al., examined PrEP knowledge, based on the awareness of the iPrEX study results and the Centers for Disease Control and Prevention’s interim PrEP guidelines for MSM, among US HIV providers in 2011 [15]. Knowledge of these two guidelines (90% and 78%) did not translate into increased PrEP prescribing (19%). We also found that ID specialists were PrEP aware but perhaps less willing to prescribe PrEP within multivariate analysis. Commonly reported concerns, the desire for more efficacy data, medication provision, and the lack of national guidelines and drug regulatory approval may explain our results. In general, future studies on provider PrEP awareness or knowledge should include standardizing the definition of these two terms, determining their relationship, and, more importantly, how they affect PrEP prescribing.

PrEP should be offered within a comprehensive prevention package [10,27,28] and physician PrEP training can optimize how to have discussions surrounding individual risk and how to promote existing, underutilized HIV prevention strategies. While we found that 83% correctly identified anal sex as the highest HIV transmission risk, this study did not assess if respondents felt comfortable engaging in sexual health conversations to correctly assess risk. PrEP workshops should incorporate discussions on how to initiate conversations about patient sexual practices using a culturally sensitive and nonjudgmental approach [29]. Furthermore, few performed regular HIV and STI testing (32% and 64%), which can be emphasized along with other prevention strategies.

Training sessions should address the PrEP prescribing concerns found in this and other studies [16, 24]. Antiretroviral resistance (91%) and risk compensation (88%) described in recent studies indicate that they are not as prevalent as previously thought [6–9,30–33]. Medication toxicity (64%) and cost (63%) have also been addressed in recent studies [34,35]. Knowledge of PrEP efficacy, patient acceptability, other countries’ use of PrEP, medication costs, and potential healthcare cost savings can set the stage for provider buy in to achieve the overall public health benefits the nation hopes to gain with PrEP rollout. We envision lessons learned from Peru’s early implementation programs, a country that recently approved TDF/FTC for PrEP, will offer regional and cultural context, which North American programs, where manufacturer-subsidized medication programs exist, cannot [36,37].

Implementation-related hypothetical situations (e.g. policy and non-policy-related) and the willingness to prescribe PrEP revealed that even if PrEP was efficacious above 90% for all at-risk groups of MSM, sex workers, and serodiscordant couples, 1 in 4 physicians in this sample would not be willing to prescribe PrEP. Further qualitative work is required to explore the qualms with biomedical HIV prevention strategies. This will be important to set the stage for implementation of future PrEP technology, such as the vaginal ring and the long-acting injectable [38–40]. Accurate dissemination among providers of high PrEP efficacy with more than 4 doses per week of TDF/FTC in clinical and pragmatic trials for MSM is important and was requested [6,41–43]. Laying out such data in comparison to efficacy of current HIV prevention options has been reported to stimulate provider integration of PrEP into preventive care [29]. We found in the scenario of having government-endorsed guidelines, only 54% of providers were willing to prescribe PrEP; thus, it will be important to see how the awareness of and the release of the WHO guidelines for PrEP 2015 [10] impact willingness to prescribe. Future work should elaborate on what national policies are needed to have the maximal effect on the willingness to prescribe. More than government-endorsed policies, providers were more willing to prescribe (60%) if medication costs were lower. Sixty-one percent of respondents were fearful of potential government funding losses for existing prevention programs. These are common concerns when resources are limited for HIV treatment and prevention [44].

Estimated HIV prevalence among MSM in Guatemala City is 12% [18,45] and provider willingness to prescribe PrEP to MSM in our study was high (77%). However, ample work remains on how to best engage MSM and other stigmatized populations with culturally appropriate HIV testing and PrEP programs, in collaboration with community-based organizations, for the goals of preventing new infections [44,46]. We found that only 55% of providers were willing to prescribe to PWID; future studies should delineate unwillingness to prescribe among certain at-risk groups.

Our study is limited by a small sample size but will help guide further provider assessments related to early PrEP implementation efforts in Guatemala. This study was administered at an urban academic public hospital and may not fully represent the different types of providers in Guatemala, which include private practice and rural-based physicians. Furthermore, our definition of “awareness” may over-report PrEP awareness when compared to other studies, which utilize specific elements of knowledge [11,15]. We anticipate the prevalence of PrEP awareness to be lower when including other provider types, different practice settings, and definitions of “awareness.”

Implementation programs should gather more rigorous baseline PrEP knowledge assessments and evaluate educational programs aimed to increase knowledge and prescribing of PrEP in Guatemala. Communication with Guatemalan medical providers indicate preliminary discussions for PrEP implementation has begun; this initiative has been spearheaded by international organizations, such as the United Nations Program on HIV/AIDS [C.R. Mejia, personal communication, January 14, 2016]. This study’s findings can be incorporated into such future discussions and within the planning of provider trainings. Documenting changes in provider awareness and willingness to prescribe over time should be on the national agenda in order to gauge successful implementation and evaluate the strategies employed.

## Conclusions

PrEP has the potential to curb HIV transmission among at-risk populations in Latin America. However, for PrEP to be implemented effectively, we must understand provider concerns and willingness to prescribe. We have provided an initial study describing physician PrEP awareness and prescribing attitudes in Guatemala to aid national and regional implementation efforts.

## Supporting information

S1 File HIV PrEP provider questionnaire for physicians, Guatemala. (DOCX)

S2 File HIV PrEP provider dataset. (XLSX)
