# Supplementary material for: Awareness and attitudes of pre-exposure prophylaxis for HIV prevention among physicians in Guatemala: Implications for country-wide implementation
Source: PLoS One. 2017 Mar 3;12(3):e0173057. doi: 10.1371/journal.pone.0173057 (PMC5336255; doi:10.1371/journal.pone.0173057)
Supplement: S1 File — (DOCX) [file pone.0173057.s001.docx]

**HIV PrEP Provider Questionnaire for Physicians, Guatemala**

| **1. What is your current age?** | ____________ in years |
| --- | --- |
| **2. Gender** | - Male - Female |
| **3. What is your current level of training?** | - Resident - Attending - Generalist - ID/HIV specialist - Other |
|  |  |
| **4. Who are the high-risk groups in Guatemala to get infected with HIV (check all that apply)?**  **Sex workers** | - Sex workers - Men who have sex with men - Prison populations - People infected with tuberculosis - Intravenous drug users |
| **5. What type of intercourse carries the highest risk of HIV transmission?** | - Anal sex - Penile-vaginal sex - Oral sex |
| **6. Which HIV prevention methods do you currently use with your patients (check all that apply)?** | - Screening for high risk sexual behaviors - Screening for intravenous drug use - STI (sexually transmitted infection) screening - Behavioral interventions (counseling) - Emphasize regular condom use - Routine HIV screening - Promotion of abstinence - Other |
| **HIV pre-exposure prophylaxis, or PrEP, is using anti-retroviral medications before expected HIV exposure, such as sexual encounters or intravenous drug use to prevent transmission. Currently, Truvada (Tenofovir/Emtricitabine) is being used for this purpose in some countries.** | |
| **7. How much do you know about PrEP?** | - Never heard of it - Know a little - Have read the studies - Have prescribed PrEP |
| **8. Have you ever prescribed HIV post-exposure prophylaxis (PEP) before? If the answer is yes, then for what purpose?** | - Never have prescribed PEP - Occupational Post-Exposure Prophylaxis (oPEP) (i.e. after a needle stick injury) - Non-occupational Post-Exposure Prophylaxis (nPEP) (i.e. after a sexual or injection drug use exposure) - Other |

**9a. If PrEP was widely available, how likely would you be to prescribe PrEP to the following populations if they are confirmed to be HIV negative?**

**“MSM” refers to men who have sex with men.**

|  | Would not prescribe | Probably would not prescribe | Neutral/  Undecided | Probably would prescribe | Would prescribe |
| --- | --- | --- | --- | --- | --- |
| MSM with multiple sexual partners, not using condoms |  |  |  |  |  |
| MSM with multiple sexual partners, using condoms regularly |  |  |  |  |  |
| Serodiscordant homosexual couple (where one partner is infected with HIV and one partner is not infected with HIV) |  |  |  |  |  |
| Serodiscordant heterosexual couple (where one partner is infected with HIV and one partner is not infected with HIV) |  |  |  |  |  |
| Female sex worker (someone who exchanges sex for money, drugs, housing or other items) |  |  |  |  |  |
| Male sex worker (someone who exchanges sex for money, drugs, housing or other items) |  |  |  |  |  |
| Intravenous drug user |  |  |  |  |  |

**9b. Of the groups described in 9a above, please circle the population to which you would be MOST likely to prescribe PrEP.**

**10a. How concerned are you with regard to the following aspects (or potential risks) of prescribing PrEP?**

|  | Not at all concerned | Somewhat concerned | Concerned | Very concerned | It would prevent me from prescribing PrEP |
| --- | --- | --- | --- | --- | --- |
| Potential side effects or toxicity of PrEP |  |  |  |  |  |
| Potential increase in high-risk behavior |  |  |  |  |  |
| Development of HIV resistance |  |  |  |  |  |
| Potential loss of federal funding to other HIV preventative measures |  |  |  |  |  |
| Cost of PrEP |  |  |  |  |  |
| Requirement for frequent follow up and testing |  |  |  |  |  |
| Other |  |  |  |  |  |

**10b. Of the options described in 10a, please circle the GREATEST concern you have about prescribing PrEP.**

**11. How would the following hypothetical scenarios affect your likelihood to prescribe PrEP?**

|  | Would not prescribe | Probably would not prescribe | Neutral/  Undecided | Probably would prescribe | Would prescribe |
| --- | --- | --- | --- | --- | --- |
| Efficacy rates for prevention were greater than 90% |  |  |  |  |  |
| Government endorses PrEP use and releases guidelines for use |  |  |  |  |  |
| Cost of medicine is decreased |  |  |  |  |  |
| Education workshops about PrEP prescribing and monitoring are provided |  |  |  |  |  |
| Other |  |  |  |  |  |

| **12. What more do you want to know about PrEP?** | - Evidence of efficacy for HIV prevention - Data concerning patient acceptability of PrEP - Other countries’ use of PrEP - Cost of PrEP - Potential health care cost savings of PrEP - Other |
| --- | --- |
